# Atypical diagnosis for typical lung carcinoid

**DOI:** 10.1186/s12890-019-0929-0

**Published:** 2019-09-02

**Authors:** Roberto Piro, Roberto Tonelli, Sofia Taddei, Alessandro Marchioni, Giovanni Musci, Enrico Clini, Nicola Facciolongo

**Affiliations:** 1 Respiratory Diseases Unit, Azienda Unità Sanitaria Locale - IRCCS Arcispedale Santa Maria Nuova, Reggio Emilia, Viale Risorgimento 80, 42123 Reggio Emilia, Italy; 2 Department of Medical and Surgical Sciences, University of Modena Reggio Emilia, Modena, Italy; 3 Pathology Unit Azienda Unità Sanitaria Locale - IRCCS di Reggio Emilia, Reggio Emilia, Italy

**Keywords:** Pulmonary carcinoid tumor, Trans-bronchial biopsy, Solitary pulmonary nodule

## Abstract

**Background:**

The diagnosis of lung typical carcinoid tumors results challenging when limited size and unfavorable sampling location is associated. It has been reported that bronchoscopy with endobronchial ultrasound (EBUS) significantly increases the diagnostic yield of peripheral nodules smaller than 2 cm.

**Case presentation:**

A 70-year-old Caucasian male complained of persistent fever and cough despite several antibiotic courses and steroid treatment. Chest radiology revealed the presence of a small single nodular opacity in the left upper lobe, whose standardized maximum uptake value (SUV) at fluorodeoxyglucose positron emission tomography-computed tomography (FDG PET/CT) was significantly high (4.5). The patient underwent bronchial endoscopy but any appreciable sign of endobronchial or intramural involvement was detected. Only radial ultrasound-guided bronchoscopy (R-EBUS) allowed transbronchial sampling whose pathological analysis revealed a typical carcinoid tumor. The patients underwent surgical lobectomy and clinic-radiological follow was started.

**Conclusions:**

With this case we aim at stressing the importance of ultrasound in the diagnostic process of lung small peripheral carcinoid, especially if they present without mucosal or sub mucosal involvement.

## Introduction

Carcinoid tumors are low-grade neuroendocrine malignancies that usually affect the gastrointestinal tract [1]. According to the mitosis number (below or above 2 mitoses/2 mm2) and the absence/presence of architectural disruptions and necrosis found on histopathology, they are classified as typical and atypical carcinoid tumors respectively [2]. Pulmonary location is reported as the second commonest site with higher prevalence in the central bronchial tract [3]. When situated in peripheral lung regions an accurate diagnosis may result more difficult due to unfavorable sampling position and often require surgical excision [4]. Furthermore these lesions are usually size-limited and highly vascularized with the bronchial mucosa that overlays the carcinoid relatively spared: these features contribute to make the diagnosis even more challenging. [5]. It has recently been reported that bronchoscopy with endobronchial ultrasound (EBUS) significantly increases the diagnostic yield in the evaluation of peripheral nodules smaller than 2 cm [6]. Here we report the case of lung peripheral typical carcinoid tumor that was not identified at the fiber optic preliminary endoscopic investigation and whose diagnosis was made possible only through radial endobronchial ultrasound-guided bronchoscopy (R-EBUS).

## Case presentation

**Fig. 1:**
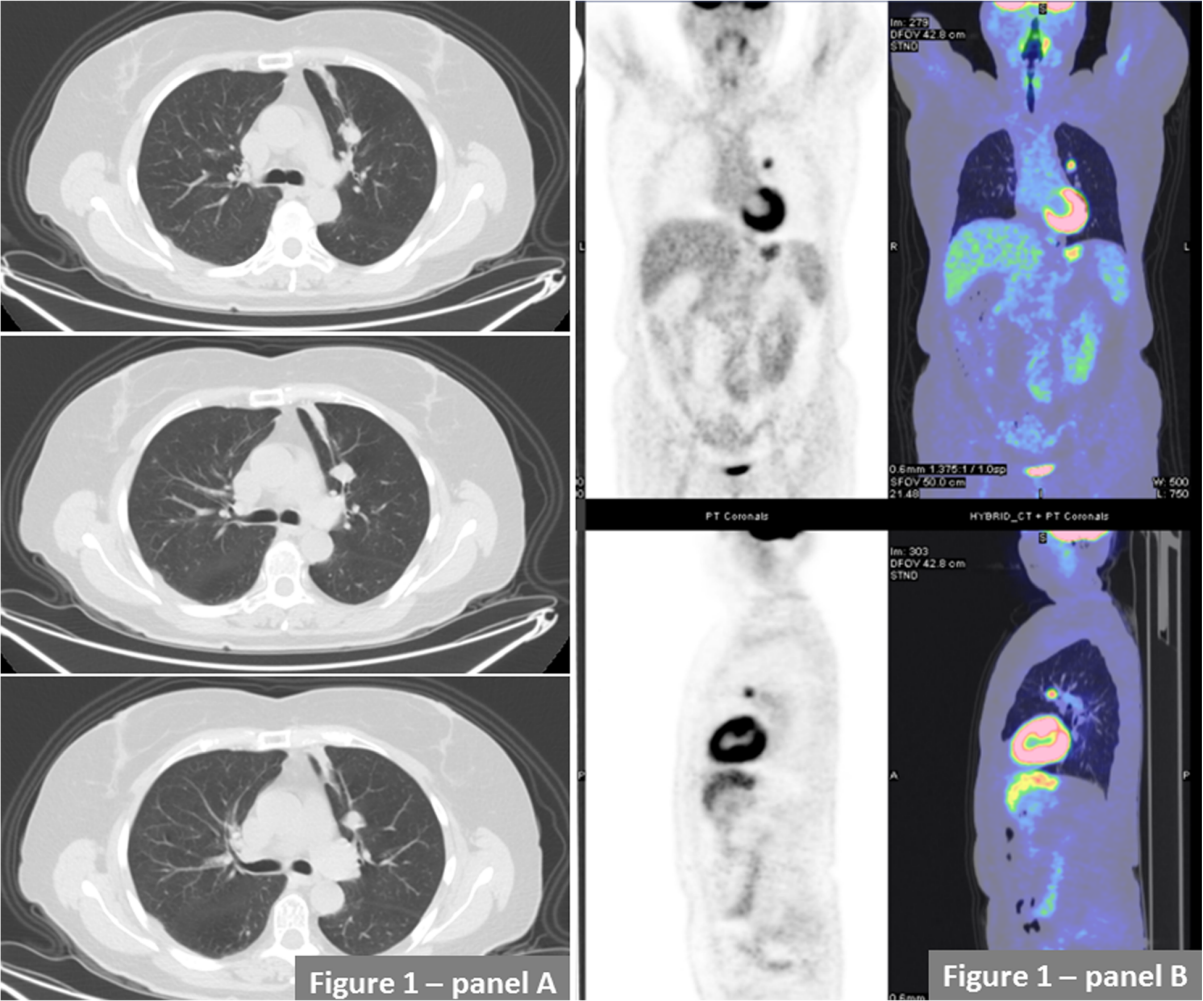
Panel **a**. Chest computed tomography (CT) scan (panel **a**) showing a single nodular opacity of 1.6 × 1.3 cm in the anterior segment of the left upper lobe. Panel **b**. Fluorodeoxyglucose positron emission tomography/computed tomography (FDG PET/CT) showing a nodular area of increased metabolic rate located in the same area of the CT-identified lung opacity

**Fig. 2:**
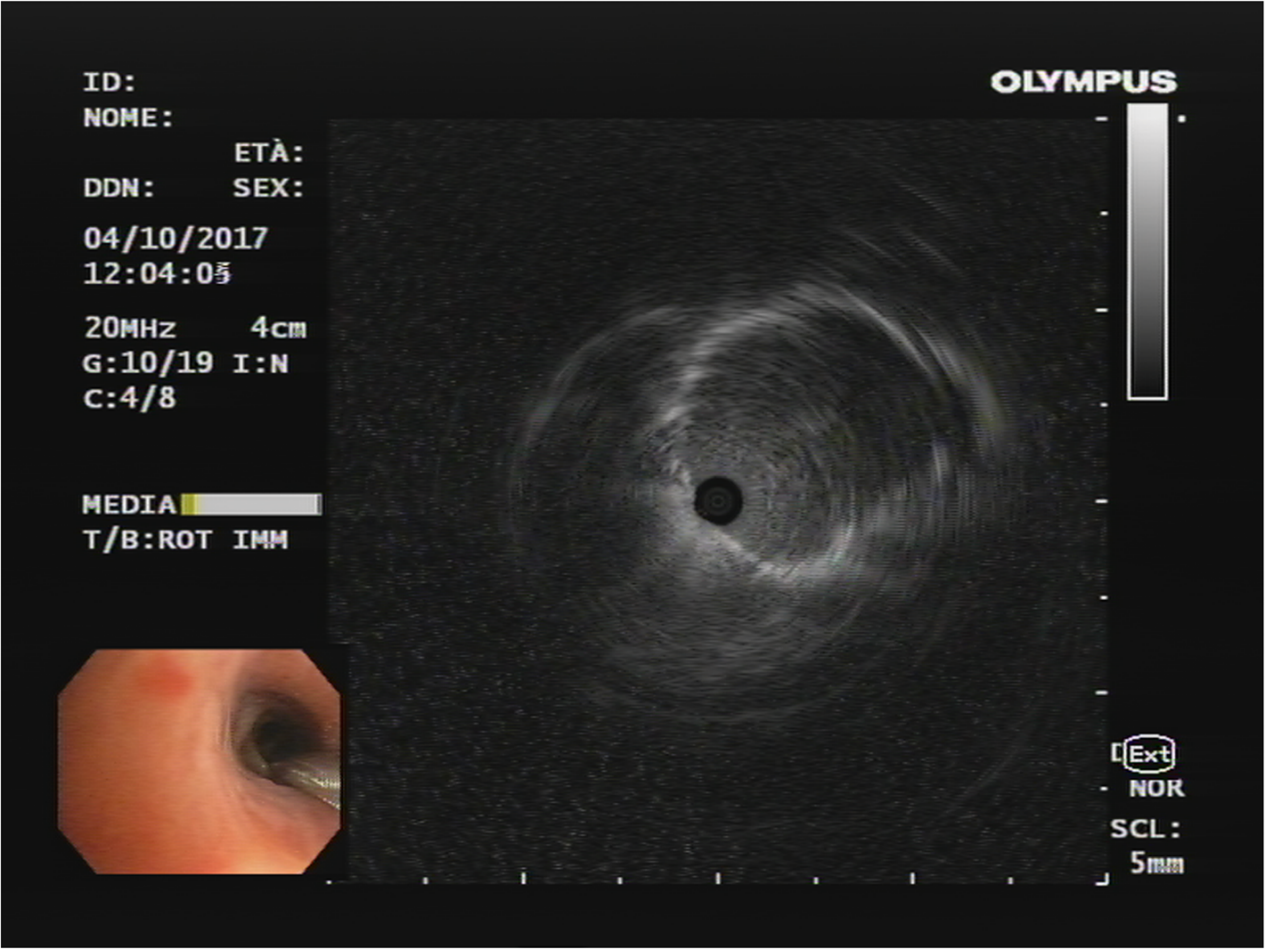
Radial probe endobronchial ultrasound image in LB3a bronchus indicating the presence of a hypoechogenic nodule from 1 ‘clock to 3 o’clock

**Fig. 3:**
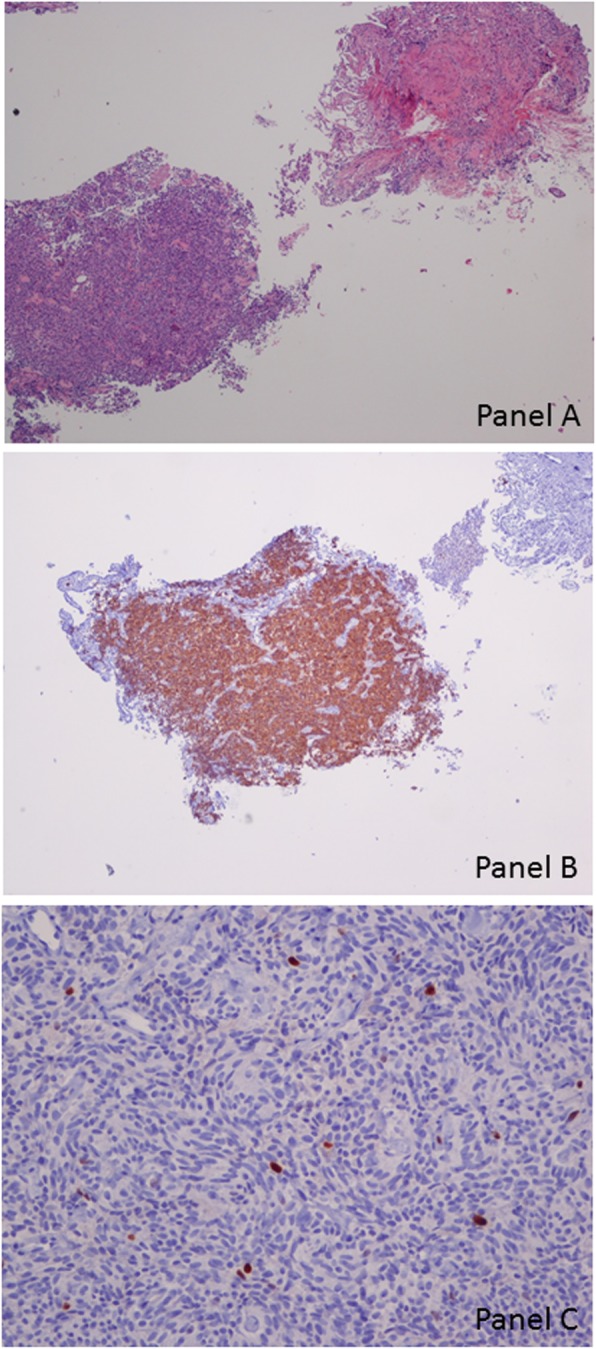
Histologic samples at different magnification of the video assisted pleural biopsy. Panel **a**. Hematoxylin-eosin stain showing fragments of unremarkable lung parenchyma and small pieces of tumor (20x). Panel **b**. Immunohistochemistry stain with chromogranin revealing a diffuse positivity among the tumor fragment. Panel **c**. Ki-67 staining showed a low proliferative activity (about 1%)

A 70-year-old mild former smoker (3 packs/year) male was admitted to the Respiratory Ward of the Santa Maria Nuova Hospital of Reggio Emilia (IT) for persistent cough and fever despite prolonged antibiotic courses and steroid therapy. Past medical history revealed surgical excision of the right vocal cord for a benign vocal cords tumor performed 30 years before. At the time of hospital admission the chest X ray showed a single lung nodular opacity of 1.6 × 1.3 cm in the left upper lobe that was confirmed by a subsequent chest computed tomography (CT) scan (Fig. 1a). The fluorodeoxyglucose positron emission tomography/computed tomography (FDG PET/TC) showed a single area of increased metabolic rate (maximum standardized uptake value (SUV) of 4.5) in the anterior segment of the left upper lobe while abdomen and brain CT scan were negative for other lesions (Fig. 1b). The patient underwent bronchial video-endoscopy (Olympus BF-H190) that did not identify any endobronchial or intramural alterations in the explorable tracheo-bronchial tree. In particular no signs of mucosal abnormalities or evidence of sub-mucosal lesions were described (Fig. 2). Thus a radial endobronchial ultrasound probe (REBUS) was necessary to identify the sub-segmental bronchus were the nodule was located and 5 trans-bronchial biopsies were performed in the apical tract of the anterior segmental bronchus of the left upper lobe (LB3a) (Fig. 2), with oval fenestrated biopsy forceps (Olympus model no. FB- 231D), without significative bleeding or other complications. Immunohistochemical investigation was broadly positive for chromogranin and synaptophysin while the proliferative index assessed by KI67/MIB1 was about 1% (Fig. 3). To complete diagnosis a 68Ga-DOTA-peptide PET/CT was also performed demonstrating a very small area of hyper-folding of the tracer (SUV max equal to 1.6). Based on these results the diagnosis of typical carcinoid of the bronchus stage cT1 cN0 cM0 was made. The patient successfully underwent surgical excision of the upper left lobe and clinic-radiological follow-up was started.

## Discussion

Pulmonary carcinoid tumors are low-grade malignant tumors of neuroendocrine origin [7] accounting for about 1% of all primary lung cancer [8]. In the last 3 decades, the incidence of lung carcinoid tumors has significantly raised, probably due to increased clinical awareness and to the improved diagnostic yield of radiologic and endoscopic procedures [9]. On CT scan peripheral carcinoid tumors usually present as a single lobulated lesion whose diameter rarely exceeds 2 cm. In some studies, 68-Gallium DOTATATE peptide PET-CT -a type of functional imaging in which a radioisotope-labeled somatostatin analog peptide binds to the somatostatin receptor found in carcinoid– has been found to improve the anatomic localization of neuroendocrine tumors [10]. Nevertheless, the small size and the unfavorable sampling features of these lesions cause that more than 30% of carcinoid tumors require thoracotomy to be diagnosed [6]. CT-guided needle biopsy might also be performed even though the diagnostic yield is moderately low and pneumothorax has been reported as a not infrequent complication for this procedure [6, 9]. When carcinoids are centrally located, bronchoscopy plays a critical role in their diagnosis, as they are visible at endoscopic evaluation [11, 12]. Generally flexible bronchoscopy is preferable; however, in patients at high risk for bleeding, rigid bronchoscopy may be indicated, both for obtaining biopsy specimens and also for performing ablation procedures [11]. If carcinoids involve the peripheral region of the lung, diagnosis results more challenging giving the difficulty to find the right tributary distal bronchial segment, and thoracoscopic resection is often the method of choice. US-guided bronchoscopy demonstrates a high diagnostic yield with low complication rate in the diagnostic evaluation of small peripheral nodules [6]. Tanaka et al. have recently reported a case of EBUS-diagnosed peripheral carcinoid tumor [11]. The authors show how the use of ultrasound technique confirmed the presence of a solid nodule located where endoscopic evaluation found a yellow sub-mucosal lesion. In our case the R-EBUS technique was essential to find the correct place to sample giving the lack of appreciable endobronchial involvement at mucosal or sub-mucosal level. It is worth noticing that peripherally radial ultrasound did not help in understanding the o’clock position of a lesion in order to guide the biopsy. However, when the lesion occupies an important portion of the bronchus lumen, this limitation could be overcome by the precise identification of the right tributary bronchus and the appropriate distance from the tip of the endoscope. When forceps are pushed in the point previously identified whit the ultrasound probe, the biopsy can be confidently performed and the rate of positivity is generally high. This limitation is greater when the lesion occupies only a small portion of the distal bronchus or when needle is used instead of forceps.

In conclusion, we report a case of a peripherally located carcinoid tumor whose diagnosis was made possible only through transbronchial biopsy performed with R-EBUS. This case emphasizes how ultrasound is more than useful in the diagnostic process of lung small peripheral tumors, particularly when they present as occult at endoscopic investigation.

## Data Availability

Not applicable.

## Abbreviations

CT: Computed tomography
EBUS: Radial endobronchial ultrasound
FDG PET/TC: Fluorodeoxyglucose positron emission tomography/computed tomography
SUV: Maximum standardized uptake value

## References

[1] Hendifar AE, Marchevsky AM, Tuli R (2017). Neuroendocrine tumors of the lung: current challenges and advances in the diagnosis and management of well-differentiated disease. J Thor Onc.

[2] Travis WD, Rush W, Flieder DB, Falk R, Fleming MV, Gal AA, Koss MN (1998). Survival analysis of 200 pulmonary neuroendocrine tumors with clarification of criteria for atypical carcinoid and its separation from typical carcinoid. Am J Surg Pathol.

[3] World Health Organization Classi cation of Tumors (2004). Pathology and genetics. Tumors of the lung, pleura and heart.

[4] Rizzardi G, Marulli G, Bortolotti L, Calabrese F, Sartori F, Rea F (2008). Sleeve resections and bronchoplastic rocedures in typical central carcinoid tumours. Thorac Cardiovasc Surg.

[5] Rosado de Christenson ML, Abbott GF, Kirejczyk WM, Galvin JR, Travis WD (1999). Thoracic carcinoids: radiologic-pathologic correlation. Radiographics.

[6] Steinfort DP, Finlay M, Irving LB (2008). Diagnosis of peripheral pulmonary carcinoid tumor using endobronchial ultrasound. Ann Thorac Med.

[7] Fink G, Krelbaum T, Yellin A, Bendayan D, Saute M, Glazer M, Kramer MR (2001). Pulmonary carcinoid: presen- tation, diagnosis, and outcome in 142 cases in Israel and review of 640 cases from the literature. Chest.

[8] Oberg K (2003). Diagnosis and treatment of carcinoid tumors. Expert Rev Anticancer Ther.

[9] Caplin ME, Baudin E, Ferolla P, Filosso P, Garcia- Yuste M, Lim E, Oberg K, Pelosi G, Perren A, Ro-ssi RE, Travis WD, ENETS consensus conference participants (2015). Pulmonary neuroendocrine (carcinoid) tumors: European neuroendocrine tumor society expert consensus and recommendations for best prac- tice for typical and atypical pulmonary carcinoids. Ann Oncol.

[10] Shell J, Keutgen XM, Millo C (2018). 68-gallium DOTATATE scanning in symptomatic patients with negative anatomic imaging but suspected neuroendocrine tumor. Int J Endocr Oncol.

[11] Tanaka A, Akamatsu H, Kawabata H, Ariyasu H, Nakamura Y, Yamamoto N (2016). Pulmonary carcinoid diagnosed by EBUS. Respirol Case Rep.

[12] Filosso PL, Rena O, Donati G, Casadio C, Ruffini E (2002). Bronchial carcinoid tumors: surgical management and long-term outcome. J Thorac Cardiovasc Surg.

